# Structure–Glass Transition Relationships in Non-Isocyanate Polyhydroxyurethanes

**DOI:** 10.3390/molecules29174057

**Published:** 2024-08-27

**Authors:** Konstantinos N. Raftopoulos, Izabela Łukaszewska, Sebastian Lalik, Paulina Zając, Artur Bukowczan, Edyta Hebda, Monika Marzec, Krzysztof Pielichowski

**Affiliations:** 1 Department of Chemistry and Technology of Polymers, Cracow University of Technology, Warszawska 24, 31-155 Kraków, Poland; izabela.lukaszewska@doktorant.pk.edu.pl (I.Ł.); paulina.zajac@pk.edu.pl (P.Z.); artur.bukowczan@pk.edu.pl (A.B.); edyta.hebda@pk.edu.pl (E.H.); krzysztof.pielichowski@pk.edu.pl (K.P.); 2 Institute of Physics, Jagiellonian University, Prof. S. Łojasiewicza 11, 30-348 Kraków, Poland; sebastian.lalik@uj.edu.pl (S.L.); monika.marzec@uj.edu.pl (M.M.)

**Keywords:** polyhydroxyurethanes, glass transition, structure–property relationships

## Abstract

The molecular dynamics, with an emphasis on the calorimetric and dynamic glass transitions, of non-isocyanate polyhydroxyurethanes (PHUs) produced by the equimolar polyaddition of polyether-based dicyclic carbonates (P-CCs) and various short diamines was studied. The diamine component consisted of a short aliphatic diamine (1,4-diaminobutane, DAB) and a more complex ‘characteristic’ diamine. The study was conducted to investigate (i) the chemical structure of the characteristic amine, (ii) its molar ratio, and (iii) the structure and molar mass of the P-CC. Infrared spectroscopy, differential scanning calorimetry, and broadband dielectric spectroscopy were employed. The P-CC, constituting the bulk of the systems, was the most crucial component for the glass transition. The characteristic amine influenced the glass transition as a result of its bulky structure, but also presumably as a result of the introduction of free volume and the formation of hydrogen bonds. The dynamic glass transition (α relaxation) trace in the Arrhenius plots showed a subtle change at a certain temperature that merits further study in the future. The charge mobility was fully coupled with the molecular mobility, as evidenced by dc conductivity being directly proportional to the characteristic frequency of α relaxation. The fluctuation in carbonyl units (β relaxation) was mildly affected by changes in their immediate environment.

## 1. Introduction

Polyurethane chemistry, traditionally understood as the reaction between an isocyanate (NCO) and a hydroxyl (OH) group, has long served as a straightforward method for the design of copolymers with tailored properties [1,2]. This versatility originates from the abundance of di- or multi-functional substances with OH and NCO groups, and the easily accessible synthesis conditions. The resulting polar urethane linkages tend to form a secondary hydrogen-bond network, leading to an easily controllable micro-phase separated micromorphology with hard microdomains consisting of urethane-rich rigid segments dispersed in a soft phase, which consists of flexible segments with some urethane ones diluted in it. Except for the composition of the system, the micromorphology properties strongly depend on the thermal history of the materials, which further enhances the possibility of tailoring their properties.

The challenge with conventional polyurethanes is the potential hazard posed by isocyanates in terms of health and safety [3,4,5]. Furthermore, the traditional reaction path can be accompanied by a number of side reactions, often associated with environmental moisture, and therefore requires strict control of the synthesis conditions [6,7]. For these reasons, much effort has been directed towards the development of non-isocyanate paths for polyurethane synthesis [4,8,9,10,11,12,13,14]. In this context, it seems that the polyaddition reaction between a cyclic carbonate and an amine group is the most promising direction [11,15,16,17]. However, this reaction does not only produce polyurethanes; a primary or secondary OH group is formed very close to the urethane linkage, attached to a nearby carbon atom. For this reason, we refer to the resulting polymers as polyhydroxyurethanes (PHUs).

The properties of conventional polyurethanes have been extensively studied over the years. The interested reader is referred to the excellent book by Prisacariu [1]. The seminal works of Koberstein’s group in the 1980s and early 1990s, along with later works by other groups, have described the relationships between the structure, composition, thermal history, micromorphology, and thermal glass transition of PUs [18,19,20,21,22,23,24,25,26,27,28,29,30,31]. On the other hand, the research on non-isocyanate polyurethanes is practically just beginning. Most works focused on the synthesis and further applications, especially in the biomedical field. Little is known about their fundamental physical properties. One could initially argue that the route of synthesis is hardly relevant to the properties of the final materials. This would be a valid assumption. However, we recall here that in non-isocyanate polyurethanes, the urethane linkage is accompanied by a hydroxyl group. This very polar group significantly hinders the ability of the system to form a microphase-separated morphology. Moreover, it provides the system with better thermal stability. In a series of articles, we also extensively reported on the remarkable hydrophilicity of these systems [32,33,34], which is attributed specifically to the presence of this hydroxyl moiety. Polyhydroxyurethanes are interesting from the point of view of special applications, such as smart coatings [8] and biomedical materials, e.g., hydrogels, drug delivery systems, and implants [4,10,11,33]. 

The molecular mobility in conventional polyurethanes, with a special emphasis on the glass transition, has been studied extensively, especially by the Vallance [35], Runt [28,29,30,31,36,37,38,39], and Pissis [27,40,41,42,43,44,45,46,47,48,49,50,51,52,53] groups. Since polyurethanes are essentially copolymers, their glass transition is generally discussed in the context of mixing laws applicable to blends and copolymers, such the Fox, Couchman–Karasz, and Gordon–Taylor laws [54,55,56]. It is generally accepted that the main glass transition in polyurethanes is that of the soft phase, with hard domains considered less mobile. As expected, mobility depends on not only the nature of the segments and their molar mass, but also on the degree of phase separation. A higher concentration of urethane groups in the soft phase signifies a slowing down of mobility, i.e., an increase in the glass transition temperature.

Less is known about the glass transition of polyhydroxyurethanes. While many works report calorimetric glass transition *T_g_* values in such systems, comprehensive studies on their dynamic glass transition are limited. In recent work, we reported the dependence of glass transition on hydrogen bonding in a linear, non-phase separated PHU system with a controlled number of hydrogen donors [57]. We showed that hydrogen bonding is the crucial parameter controlling mobility. Charge mobility was found to be strongly coupled to segmental mobility, in terms of dynamics but also magnitude. In another work, we studied the molecular dynamics in a cross-linked system covalently modified with polyhedral oligomeric silsesquioxanes (POSSs). In this system, however, the phenomena affecting the glass transition were related to the effects of nanoaggregates and alterations in the polymeric topology [34].

Given the complexity of the phenomena affecting molecular mobility, it is evident that a study on the effects of the nature of the components and their proportions in the system, as well as the size of segments in polyhydroxyurethanes, is due. 

The objective of the presented research was to provide an understanding of the relationships between the structure of the polyhydroxyurethane macrochains and their molecular mobility. The work at hand focused on the dependence of the glass transition on the chemical structure of the polyhydroxyurethanes with respect not only to the different types of amines and cyclic carbonates, but also with respect to the molar ratios of the substrates. Special attention was paid to the thermal and dynamic glass transitions and long-range charge mobility. Differences in molecular mobility were explained in terms of macrochain interactions that were studied here using infrared spectroscopy. 

**Scheme 1:**
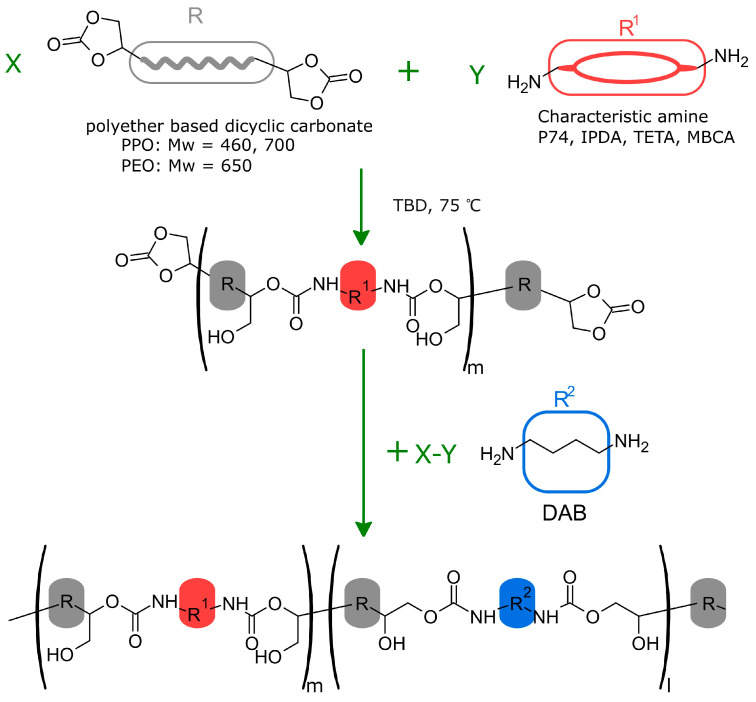
Synthesis process. Abbreviations are explained in the text and in Table 1.

The systems under investigation were linear and based on difunctional cyclic carbonates with a polyether chain. The details of the synthesis are reported in Section 4 and shown in Scheme 1. The polyethers react with a mixture of two amines equimolar to the polyether. In the first stage, the polyethers react with a selected complex amine (called the ‘characteristic amine’ in the following) at a ratio denoted as “X:Y” in which, X refers to the mols of the cyclic carbonate and Y to the mols of the characteristic amine. In the second step, 1,4-diaminobutane (DAB) is added to the system at a molar fraction of X:X-Y to form a linear system. 

Here, three partially overlapping groups of materials were studied (Table 1).

In the first group (Group I), the characteristic amine varied between the materials, while its molar fraction and the other components of the system were the same. Four amines were used: (i) 4,4′-methylenebiscyclohexanamine (MBCA), which consists of two six-membered alicyclic rings; (ii) Priamine 1074^TM^ (P74), which has a bulky structure centered on an alicyclic six-membered ring; (iii) isophorone diamine (IPDA), another six-membered alicyclic amine that is less bulky; and (iv) triethylene tetramine (TETA), a short linear amine bearing two secondary amine groups, which are known to enhance hydrogen bonding in PHUs [33,57].

The materials of the second group (Group II) varied with respect to the molar fraction of the characteristic amine, which in this case was MBCA. The polyether-based cyclic carbonate (P-CC) used was the same in all materials.

In the third group (Group III), the P-CC was varied in the systems based on MBCA (as the characteristic amine) at a molar ratio of 2:1. The P-CC used in Groups I and II [poly(propylene oxide, PPO, with nominal *M_w_* of 460] was compared to a P-CC with a longer PPO (nominal *M_w_* of 700), and a P-CC based on poly(ethylene oxide) (PEO) that had a nominal *M_w_* of 650.

In summary, the free parameters of this work were as follows: (I) the chemical nature of the characteristic amine, (II) the molar ratio of the cyclic carbonate to the characteristic amine, and (III) the nature and size of the polyether backbone of the P-CC, which actually constituted most of the mass of the system. Before proceeding to the study of molecular and charge mobility, we tried to understand to some extent how hydrogen bonding is affected by these parameters. Then, we studied the calorimetric glass transition temperature, and finally the dynamic glass transition, and the charge transport properties of the systems using broadband dielectric spectroscopy (BDS). Special attention was paid to the fragility (cooperativity) of the dynamic glass transition and the coupling between molecular and charge mobility.

Taking into account the high hydrophilicity of the samples and the strong impact of absorbed moisture on molecular mobility [32,33,34], the materials were studied after a two-step drying process: the first drying step was carried out in a vacuum oven at 100 °C, and the second step involved conditioning the samples over phosphorus pentoxide to remove any residual humidity.

## 2. Results

### 2.1. Structure—Hydrogen Bonding

**Figure 1:**
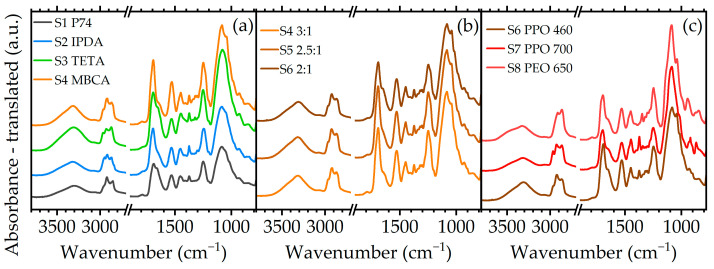
FTIR spectra of all materials under investigation. (**a**) Series I, (**b**) series II, and (**c**) series III. Note that certain samples belong to two series and that the scale is different before and after the *x*-axis break.

Figure 1 shows the FTIR spectra of all the materials. The residual band of the cyclic carbonate rings (1800 cm^−1^) in the materials’ spectra was of negligible intensity, so a high conversion rate and therefore high molar mass (above the entanglements limit) was assumed. The spectra are qualitatively similar to those reported in systems prepared in a similar fashion [57,58]. Starting from the side of high wave numbers, the broad peak around 3300 cm^−1^ contained contributions from the stretching of OH and NH groups [59,60], and a complex band around 2900 cm^−1^ was related to the stretching of C-H bonds [61]. The 1900–1600 cm^−1^ region is more interesting since it contained mainly contributions from the stretching of carbonyl groups, either on unreacted CC groups (weak peak around 1800 cm^−1^) [62] or on the urethane group (complex band centered around 1700 cm^−1^, often named as ‘amide I’) [63]. A sharp peak around 1530 cm^−1^ also corresponded to the urethane group and more specifically to the bending of N-H bonds and stretching of C-N bonds [64]. Next to it, two weaker peaks at 1450 and 1375 cm^−1^ were related to the bending of C-H bonds [60,65]. The prominent peak around 1255 cm^−1^ was related to the bending of the N-H bonds and stretching of the C-N bonds in the urethane group [64]. The very complex and intense band around 1100 cm^−1^ mainly contained contributions from the stretching of various C-O bonds, which were abundant in the materials at hand (ether, cyclic carbonate, and urethane) and their various vibrations [65]. At lower wavenumbers, rocking vibrations of C-H groups could be observed as weak signals [61].

A detailed investigation of the effects of the structure on the spectra was beyond the scope of this research. However, we were interested in the hydrogen bonding, which can have a marked effect on the calorimetric and dynamic glass transitions. In previous works by us [32,33,34,57,58,66,67] and others [68,69,70,71,72], the hydrogen bonding in polyurethanes was assessed on the basis of the aforementioned amide I region of the spectra (1600–1750 cm^−1^). This is a complex band containing contributions from non-hydrogen bonded carbonyls (centered at ~1720 cm^−1^), ‘weakly’ or ‘single’ hydrogen-bonded carbonyls (centered at ~1700 cm^−1^), and ‘strongly’ hydrogen-bonded carbonyls (centered at ~1660 cm^−1^). In our previous work, we showed that the intensity ratio of strongly bonded carbonyls correlated strongly with the glass transition temperature and other properties of the system (water absorption capacity, Young’s modulus, etc.) in a system where only the composition of the amine component varied [57].

**Figure 2:**
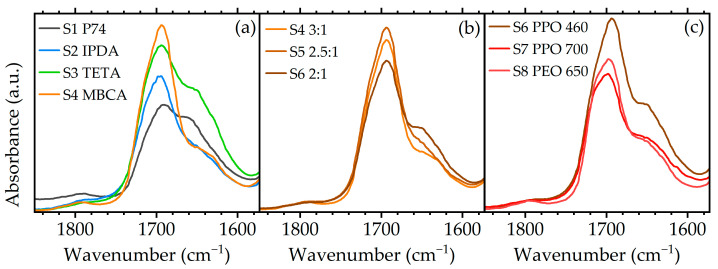
FTIR spectra of all materials in the amide I region related to the carbonyl of the urethane group. (**a**) Series I, (**b**) series II, and (**c**) series III. Note that certain samples belong to two series.

From the raw spectra, it is obvious that within series I (Figure 2a), the systems based on amines P74 and TETA showed the highest ratio of strongly hydrogen-bonded carbonyls. For the TETA system, this was attributed to the hydrogen bonding of carbonyls on the NH groups of TETA [57]. The high ratio of strongly bonded carbonyls in the P74 system is at first glance counterintuitive, since the long ligands of the amine should, in general, cause steric hindrance to the formation of hydrogen bonds. The ligands bearing the reactive groups, i.e., the ligands that are part of the main chain, are quite long, allowing for the arrangement of chains in a way that is favorable for hydrogen bonding. The remaining alicyclic amines (MBCA and IPDA), in general, showed much less hydrogen bonding, which should be attributed to the rigidity of the cycloaliphatic structures in a quite short distance from the urethane groups.

With the increasing ratio of MBCA in the system (series II, Figure 2b), interestingly, the intensity ratio of the peak related to strong hydrogen bonding was similar in the materials with molar ratios of 3:1 and 2.5:1 but it increased significantly when the molar ratio became 2:1. An increase is justified by the higher concentration of MBCA–hydroxyurethane segments. The initial stability indicates that synergy between MBCA and DAB amines plays a role in the degree of hydrogen bonding. This was also the case in an earlier system where the molar ratio of TETA was the free parameter [57]. The nature of this synergy is not yet understood; however, it could be merely related to the kinetics of the reaction and gradual equilibration of the system.

The molar mass and structure (PPO/PEO) of the polyether CC (series III, Figure 2c) did not have a marked effect on the ratio of strongly hydrogen-bonded carbonyls. This is counterintuitive, at least for the dependence on molar mass, as a shorter chain would facilitate the association of urethane units. The reason behind this needs to be clarified in future work.

### 2.2. Thermal Glass Transition

**Figure 3:**
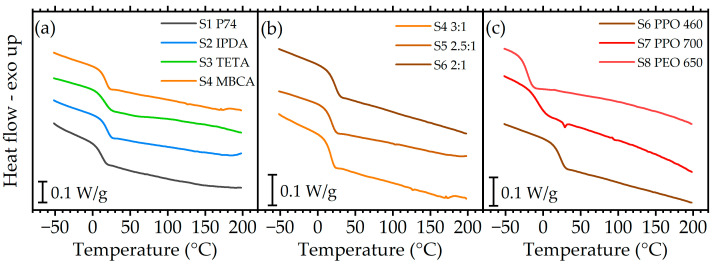
Normalized DSC curves for all materials under investigation. (**a**) Series I, (**b**) series II, and (**c**) series III. Note that certain samples belong to more than one series. The curves have been translated for clarity.

The only thermal event in the DSC curves in the range of 50–200 °C was a step corresponding to the thermal glass transition. No melting or other phase transitions were observed (Figure 3). This means that the systems at hand were fully amorphous, which is explained by the fact that the polyether chains are not long enough to form long-range order themselves (e.g., in the form of crystallites) [35,48]. Furthermore, the urethane units appear on the chain in pairs separated by the amine-derived block, and do not form long urethane-rich segments which could eventually lead to microphase separation, as is the case in conventional polyurethanes [18,19,22]. In addition, the OH groups in the vicinity of the urethane groups are also expected to hinder ordering in PHUs compared to conventional PUs.

Starting from series I, the material based on amine P74 had the lowest glass transition temperature *T_g_* at 10.8 °C, and the highest heat capacity change Δ*c_P_* (Table 1). This is likely due to the long side chains that increase the free volume and thus facilitate mobility in the system. The systems with the simpler TETA and MBCA amines had similar glass transition temperatures, with the MBCA-based one showing a slightly higher *T_g_*, presumably because of its alicyclic, bulkier (and thus less mobile) structure. Interestingly, the IPDA-based system was significantly less mobile (highest *T_g_*). The values of *T_g_* did not correlate with the degree of hydrogen bonding, as discussed in Section 2.1. This shows that the mobility restrictions posed by the rigidity of the amine overshadowed the effect of hydrogen bonding.

The ratio of MBCA in the system (series II) also did not have a significant impact on the glass transition temperature in this limited range of compositions.

The polyether chain was the factor that had the strongest effect on the glass transition temperature. Comparing the two molar masses of PPO-CC, the material based on the longer one had a *T_g_* approximately 20 K below that of the material based on the shorter one. This can be understood in two ways, both related to the mass fraction of the polyether and amine segments. The shorter the polyether chain, the higher the content of amine-derived segments, which have, on either side, a quite rigid hydroxyurethane group. On the one hand, these segments, especially the MBCA ones, are quite bulky and rigid, and on the other hand, they can form hydrogen bonds with other similar segments, leading to a physical network. Both mechanisms slow down mobility and thus increase *T_g_*.

Between the polyether-CCs with similar molar masses and structures, the PHU based on PEO exhibited a *T_g_* that was much lower than that of the PPO-based one. This is somewhat unexpected as the PPO chain is more mobile (with a *T_g_* of ~ −70 °C in the region of high M_w_ [73]) than PEO (corresponding *T_g_* is ~ −52 °C [74]). Given the similar molar masses of the two polyether chains, the concentration of the MBCA–hydroxyurethane segments is similar between the two materials and thus the observation cannot be attributed solely to mixing effects. The more radical slowing down in PPO should thus be attributed to different interactions between the amine and polyether or to differences in chain packing. Moreover, one should bear in mind that previous measurements of *T_g_* of PEO were conducted in very crystalline samples, while here, the materials were fully amorphous and thus significantly more mobile.

### 2.3. Overview of Dielectric Spectra

**Figure 4:**
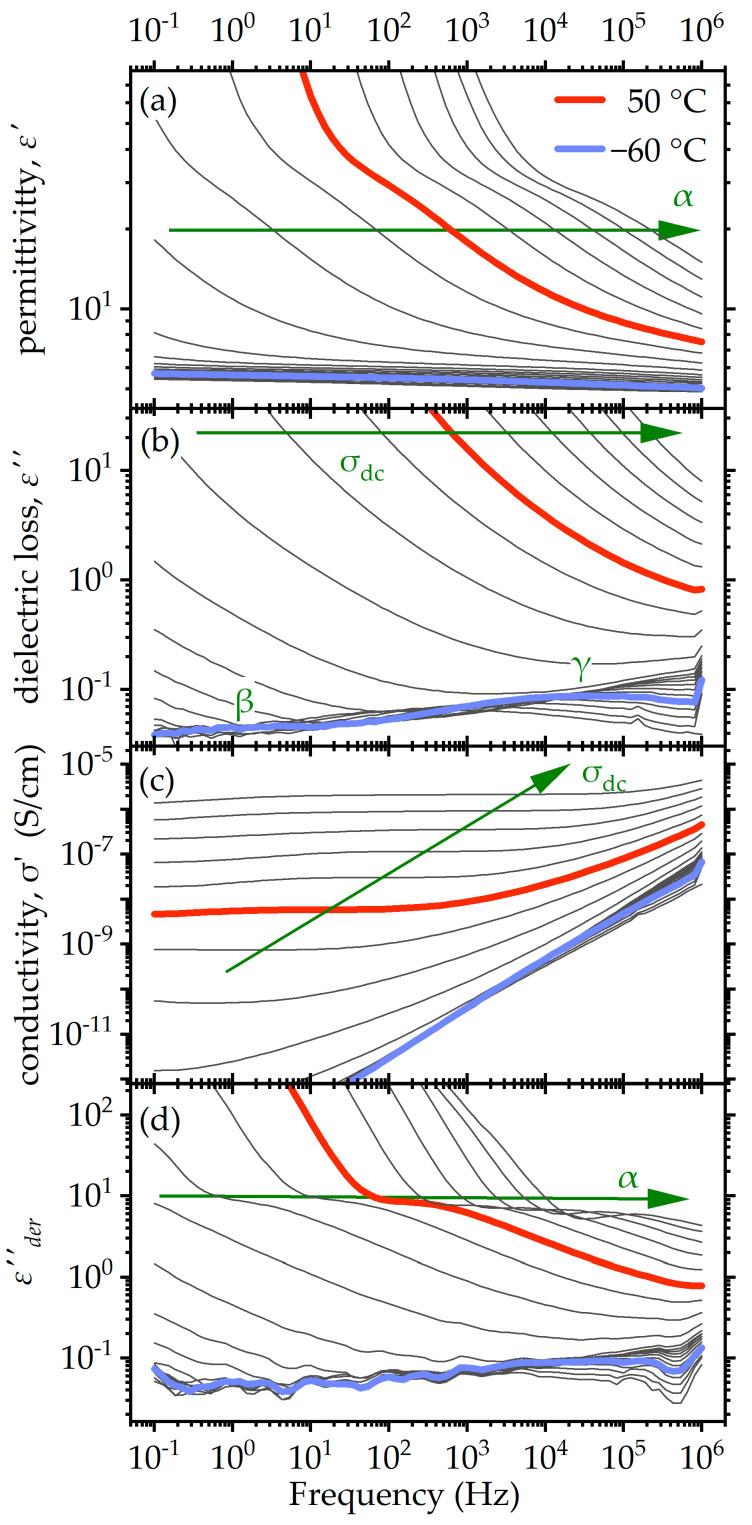
Dielectric spectra of sample S4 in the four different formalisms discussed in this article. Data are shown as a representative case. (**a**) Real part of the dielectric function *ε*′; (**b**) imaginary part of the dielectric function *ε*″; (**c**) real part of the complex conductivity *σ’*; (**d**) “conductivity free” *ε*″ approximated according to the methodology of Wübbenhorst and van Turnhout [78] (details in Section 4.5). Spectra are shown in steps of 10 K. Spectra at two key temperatures are highlighted, and the arrows show the progress of key features of the spectra.

Before proceeding to the study of the effect of the structure on the electrical and dielectric properties, it is necessary to give a short description of the raw spectra and the phenomena observed in them. All materials showed the same phenomena. They will be discussed using S4 as a representative example (Figure 4).

Starting from low temperatures, the γ relaxation was visible as a weak peak on the high-frequency side of the *ε*″ spectra (Figure 4b). This relaxation is associated with the crankshaft motion of methylene sequences and is very often observed in polyurethanes and other polyether-containing systems [40,53,75,76]. It was followed on the low frequency side by a weak (barely visible in the current scale) peak corresponding to the β relaxation. In polyurethanes, β relaxation is associated with the mobility of the carbonyl group, with the attached water molecules acting as probes [27,50,77]. It is known to be much weaker in dry polyurethanes, as is the case here, or even completely absent [75]. However, similar relaxations are often also observed in oligomeric systems with polar terminal groups such as NH_2_ [76] or OH [48], even in dry form.

The α relaxation, associated with the dynamic glass transition appears as a step in the *ε*′ spectra at higher temperatures (Figure 4a). In the *ε*″ spectra (Figure 4b), it is completely masked by the dc conductivity, manifesting itself as a very strong contribution on the low frequency side. To analyze it effectively, we followed an approach proposed by Wübbenhorst and van Turnhout [78], and calculated the “conductivity free” *ε*″(*f*) (Figure 4d), as described in Section 4.5. In this representation, the steps are converted into peaks. However, here, the peak was not fully formed due to a rapid increase in *ε*′(*f*) toward low frequencies. This upturn is related to long-range charge trapping, especially at the interfaces with the electrodes (electrode polarization). It is a parasitic phenomenon, and for this reason, we ignored this area of the spectra. In any case, the derivative spectra are clear enough for a meaningful analysis by model fitting, as we will describe later in the text.

The real part of conductivity *σ’(f)* (Figure 4c), as expected, with increasing temperature, formed plateaus of increasing values, corresponding to the dc conductivity *σ_dc_*. A slight downturn at lower frequencies was related to electrode polarization.

**Figure 5:**
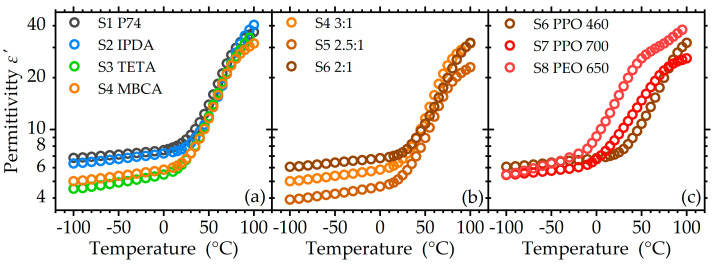
Isochronal plots of *ε*′ at a high frequency (10 kHz) that are not affected by electrode polarization. (**a**) Series I, (**b**) series II, and (**c**) series III. Note that certain samples belong to two series.

To evaluate the dependence of polarizability with temperature, we replotted the *ε*′ values recorded at 10 kHz as a function of temperature (Figure 5). This frequency was high enough to be unaffected by electrode polarization in the whole temperature range. The most prominent feature of the curves was a step corresponding to the α relaxation associated with the glass transition. A barely visible step at –60 °C was associated with γ relaxation.

Interestingly, the characteristic amines that provided higher polarizability were the P74 and IPDA ones (Figure 5a), i.e., those that bear side groups, allowing for more free space for charge separation. On the contrary, TETA, despite bearing polar groups, had the lowest polarizability, especially in the glassy state, which should be attributed to a tighter packing, which is expected due to its linear topology, and possibly due to intermolecular interactions.

The characteristic amine mol ratio, in the case of MBCA, did not have a monotonic influence on polarizability (Figure 5b). However, there seemed to be a positive qualitative correlation between polarizability and the fraction of strongly bound carbonyls (Figure 2b). The type of polyether backbone of the CC (Figure 5c) did not have a significant influence on the polarizability in the glassy state (i.e., at temperatures below the onset of α relaxation). The slightly higher value for the material based on a lower *M_w_* PPO backbone should be attributed to the higher concentration of polar hydroxyurethane groups. In the rubbery region, there was a dependence, which was related to the strength of the segmental relaxation α.

### 2.4. Local Mobility—β and γ Relaxations

**Figure 6:**
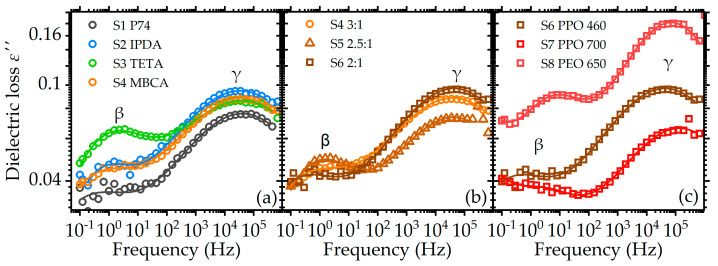
Comparative dielectric loss spectra at –60 °C, where both secondary relaxations are visible. (**a**) Series I, (**b**) series II, and (**c**) series III. Note that certain samples belong to two series. For clarity, only every second point is drawn. The lines are the fits of two Cole–Cole terms.

As commented earlier, β relaxation in polyhydroxyurethanes was very clearly visible, even though the materials had been carefully dried (Figure 6). Interestingly, among the characteristic amines (series I, Figure 6a), the best relaxation was provided by TETA, which points to the contribution of NH groups to the relaxation. This is in agreement with the presence of a relaxation with similar dynamics in poly(propylene oxide) diamine [76]. A strong and quite fast β relaxation was also visible for the material based on PEO and MBCA (series III, Figure 6c). In this case, it was also somewhat faster than that of the PPO-based materials.

The type of amine (series I, Figure 6a) did not appear to have a significant influence on the γ relaxation except for a slightly lower intensity for the material based on the amine P74, which can be simply explained by the smaller concentration of polyether in the system due to the large molar mass of the amine. This indifference of the γ relaxation dynamics to the amine points to very limited interactions between carbonyls and the polar groups around the amine-derived segments. Unexpectedly, the material based on the shorter PPO chain had a stronger relaxation than that of the material based on the longer polyether. The γ relaxation of the PEO-based material, as expected due to the longer methylene sequences, was stronger than that of its PPO-based counterparts.

**Figure 7:**
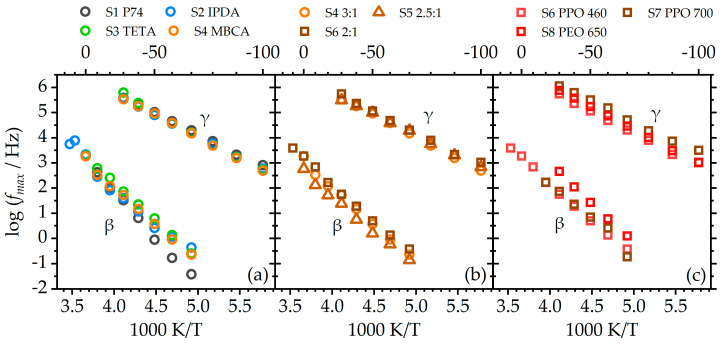
Arrhenius maps for secondary relaxations for all materials under investigation. (**a**) Series I, (**b**) series II, and (**c**) series III. Note that certain samples belong to two series.

To study the secondary relaxations in detail, a sum of two Cole–Cole terms was fitted to the data (Figure 6), as described in Section 4. The quality of the fit was excellent. The results on a time scale are shown in the Arrhenius plot in Figure 7.

**Figure 8:**
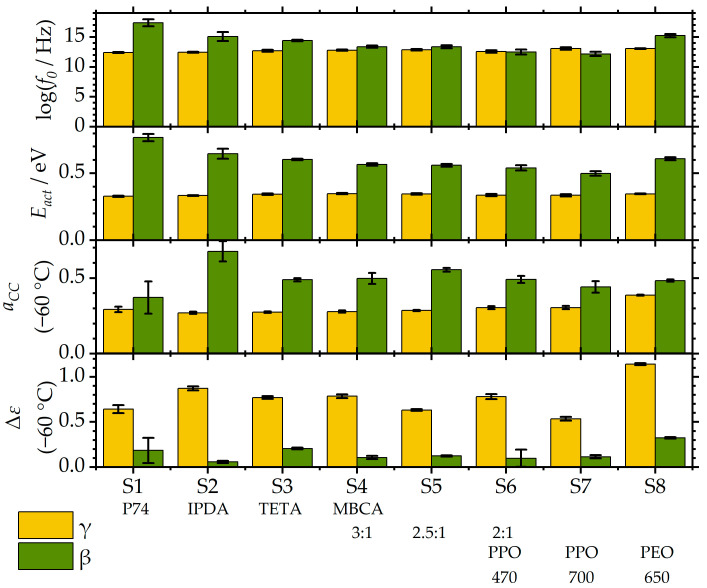
Characteristic parameters of the two secondary relaxations, which were dependent on the structure.

As expected for local relaxations, the traces followed the Arrhenius law. γ relaxation was not significantly affected by the characteristic amine nor by its molar fraction. However, the polyether seemed to play a minor role in the dynamics. This is expected since γ relaxation originates from methylene sequences on the polyether. The longer PPO and PEO seemed to exhibit slightly accelerated dynamics. This was also confirmed by fitting using the Arrhenius law:

(1) $$f_{\max}=f_{0}e^{-\frac{E_{act}}{kT}}$$

where $f_{0}$ is the phonon frequency, $E_{act}$ is the activation energy, and k is the Boltzmann constant. The results shown in Figure 8 confirmed that there were no significant changes in these parameters for γ relaxation when any of the parameters of this study were changed.

All materials based on PPO-CC had the same broadening exponent *a* when measured at −60 °C (Figure 8c). A somewhat higher *a* exponent was observed for the material based on PEO (S8), indicating a narrower distribution of relaxation times.

The strength Δ*ε* of the relaxation varied with the three studied parameters (Figure 8). Interestingly, it did not correlate with the mass of the polyether in the system. The reason for this observation is, at this point, not understood.

The dependence of the dynamics of β relaxation on the polymer structure was more complex than that of γ, as can be seen both in the Arrhenius plot in Figure 7 and in the parameters reported in Figure 8. At this point, we would like to refrain from further comments on these values; however, we would like to note that variations in the activation energy of β relaxation in the polymer structure and packing have been previously reported in conventional and hyperbranched polyurethanes [77,79].

### 2.5. Dynamic Glass Transition—α Relaxation

**Figure 9:**
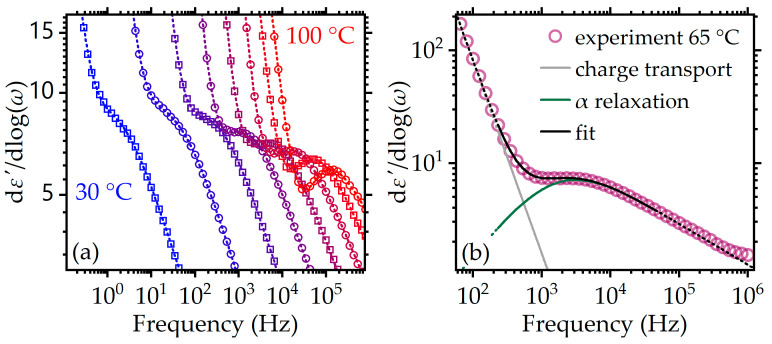
(**a**) Derivative spectra of the representative sample S4 in steps of 10 K, in the temperature region where α relaxation was visible in the experimental window. Lines are fits of the employed model; the details are provided in the text. (**b**) A detailed example of the fitting of the model on the spectrum recorded at 65 °C.

The peaks corresponding to α relaxation were generally clearly visible only at high temperatures (Figure 9a). At lower temperatures, α relaxation was only visible as a shoulder on the slope of the charge transport phenomena. A derivative of the Havriliak–Negami equation for the α relaxation peak along with a power law accounting charge transport effects describes the spectra well (Figure 9b); however, at lower temperatures, the results on the asymmetry exponents were somewhat skewed due to the lack of a clear low-frequency slope on the peak.

**Figure 10:**
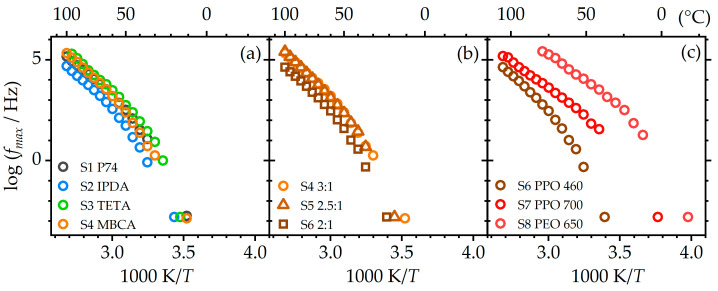
Arrhenius plots for all materials under investigation. Calorimetric glass transition temperatures (*T_g_*) are plotted at an equivalent relaxation time of 100 s. The calorimetric *T_g_* values for P74 and MBCA in panel (**a**) have been intentionally slightly translated vertically for visibility. (**a**) Series I, (**b**) series II, and (**c**) series III. Note that certain samples belong to two series.

The time scale of α relaxation was quantified as the peak frequency $f_{\max}$ and is plotted against inverse temperature in the Arrhenius plot in Figure 10. It is interesting to note that the traces did not follow a uniform Vogel–Fulcher–Tammann behavior, but for some of them, there was a subtle change in the slope in roughly the kHz region. The reason behind this observation is not clear at this point, but it could be related to a structural transition. We will return to this point in the discussion section. Overall, the traces corresponded very well to the calorimetric glass transition temperature plotted at the frequency corresponding to an equivalent relaxation time of 100 s.

The most influential parameter, regarding the time scale of the dynamic glass transition, was the nature and molar mass of the P-CC component (series III, Figure 10c). Consistent with the data for calorimetric glass transition, the PEO-based system had a much faster dynamic glass transition than its PPO-based counterparts. The system based on the longer PPO polyether showed a faster relaxation than the system based on the shorter PPO polyether, especially at low temperatures; but, because of the change in the slope mentioned earlier, the two relaxations approached each other at higher temperatures.

The concentration of the characteristic amine MBCA had a smaller effect on the α relaxation dynamics (series II, Figure 10b). Only the material with a higher concentration seemed to have a slight slowing down effect, which should be attributed to the bulkier and thus less mobile structure of the MBCA compared to the DAB one.

The type of characteristic amine also did not have a pronounced effect, especially at higher temperatures (series I, Figure 10a). However, it was evident that the IPDI-based material had somewhat slower dynamics.

**Figure 11:**
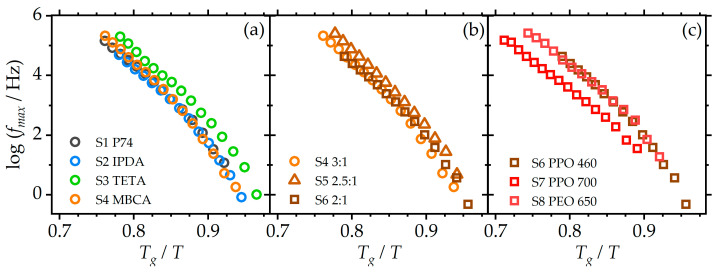
Angel plots for all materials under investigation. (**a**) Series I, (**b**) series II, and (**c**) series III. Note that certain samples belong to two series.

Traditionally, the cooperativity of the relaxations is evaluated on the basis of the fragility/strength parameters obtained by fitting the Vogel–Fulcher–Tammann equation to the data. In the case at hand, however, this was not possible due to the aforementioned change in curvature in the kHz region. For this reason, we resorted to a model-free approach based on so-called Angell plots, i.e., the logarithm of the peak frequency of the α relaxation peaks as a function of the ratio of $T_{g}$ and temperature (Figure 11) [80,81]. In these plots, a more concave trace, located towards lower temperatures and higher frequencies, pointed to a more cooperative relaxation. In that respect, it can be observed that the changes in cooperativity were rather subtle. The materials that tended to deviate were the TETA-based one in series I (Figure 11) and the material based on the longer PPO carbonate in series III. The reason for this is unclear.

For completeness, in Table 2, the strength Δ*ε* and the shape exponents of α relaxation are shown, which were determined using the fitting procedure at the representative temperature of 65 °C, and a clear peak was visible in the spectra of all materials. In series I and II, Δ*ε* did not correlate with the strength of the calorimetric glass transition, as expressed by the Δ*c_P_* value (Table 1). This is to be expected because of the vastly different chemical structures and thus polarizability of the polymeric segments. However, there was a correlation in series II. The broadening exponent *a* had similar values for all PPO-based PHUs but was significantly higher for the PEO-based one, indicating a higher dynamic homogeneity. This indicates that more polar PEO segments mix with polar hydroxyurethane moieties in comparison with the more hydrophobic PPO segments. The asymmetry exponent *b* varied significantly between materials. Most notably, the PEO-based material showed a quite low value compared to its PPO counterparts, indicating a distribution of relaxation times skewed on the faster modes side.

### 2.6. dc Conductivity Coupling with Molecular Mobility

**Figure 12:**
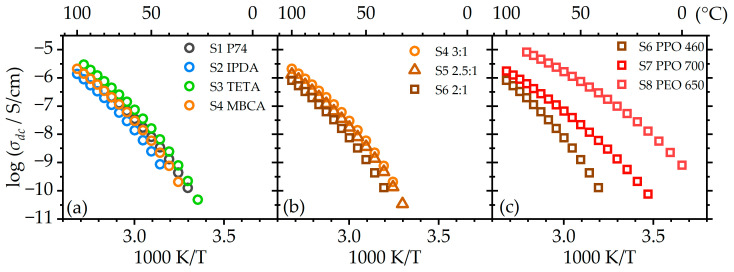
Arrhenius plot of dc conductivity for all materials under investigation. (**a**) Series I, (**b**) series II, and (**c**) series III. Note that certain samples belong to two series.

The dependence of the dc conductivity of the materials on temperature (in the Arrhenius plot) followed a clear Vogel–Fulcher–Tammann behavior (Figure 12), as opposed to the deviation observed for the α relaxation behavior (Figure 10). However, it was also evident that the trends were the same, pointing to a coupling of the segmental and charge mobility.

**Figure 13:**
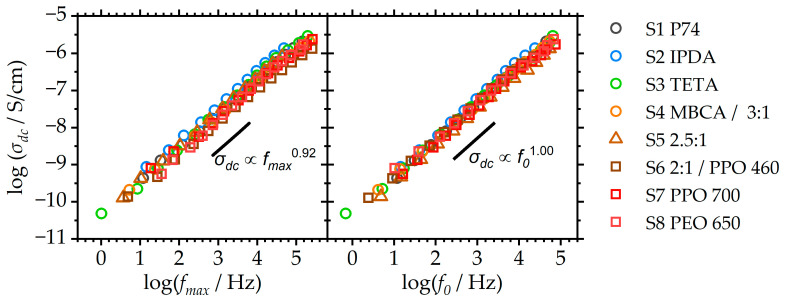
Correlation of the time scale of molecular dynamics and dc conductivity for all materials under investigation. In panel (**a**), dc conductivity is plotted against the maximum frequency and in panel (**b**) against the characteristic Havriliak–Negami frequency.

In order to further strengthen this point, in Figure 13, we plot the logarithm of dc conductivity *σ_dc_* as a function of the characteristic frequencies of the α relaxation, i.e., the maximum *f*_max_ of the peak and the characteristic frequency *f*_0_ of the Havriliak–Negami model. In both cases, the traces collapsed into one master curve, the observation being more pronounced when *σ_dc_* was plotted against *f*_0_. The master curve in both cases followed a power law. From the collective fits of all the samples with power laws, it was found that the exponent in the case of *f*_max_ was 0.92 ± 0.02 and—more interestingly—the exponent for *f*_0_ was practically unity (1.00 ± 0.01), i.e., conductivity was directly proportional to the characteristic frequency of α relaxation. Such a straightforward scaling has also been observed in TETA-based systems; however, varying the TETA content had a minor influence on the magnitude (pre-exponential factor) of conductivity.

## 3. Discussion

The discussion will focus on the most interesting findings of this work, i.e., the ones regarding the segmental mobility glass transition and its relation to charge mobility. We will also explicitly state the questions raised by the work at hand.

From the results presented and partially discussed so far, it was evident that the glass transition, both in its thermodynamic and dynamic manifestation, was mostly controlled by the type of polyether segments (Table 1). This is to be expected as this is the most abundant component in the system. Even at a first approximation, ignoring enthalpic interactions between segments, standard mixing models such the Fox [54], Couchman–Karasz [55], and Gordon–Taylor [56] models predict that in copolymers, the glass transition temperature behavior is a rather ‘smooth’ function of the fraction of each type of segment in the system. However, an unexpected result in the current work was that, although PEO had a higher *T_g_* than PPO by ~20 K, their polyhydroxyurethane copolymers with the same amines showed opposite behaviors despite the similar molar masses of the polyethers. This means that the mobility of PPO is much more affected by the presence of the amine-derived segment and the associated rigid hydroxyurethane linkage. The large difference cannot be explained solely by the small differences in the mass fraction of the amine-derived segments in the two systems. It could be argued that the *T_g_* values of PEO reported in the literature were unavoidably measured in semicrystalline systems, where the mobility of the amorphous phase is expected to be somewhat slowed down compared to the fully amorphous systems studied here; however, crystallinity imposes changes in *T_g_* on the order of several degrees and not several 10 K as was the case here [82]. Therefore, the answer should be sought in the intersegmental synergistic effects between the ether and amine segments. An obvious thought is that the two polyethers have very different behaviors with respect to interaction with polar units since PEO exhibits a hydrophilic nature, while PPO is rather hydrophobic. However, on the one hand, the FTIR spectroscopy results did not provide evidence for vast differences in hydrogen bonding on the polyethers (Figure 1), and, on the other hand, the hydrophilicity of PEO should signify stronger interactions with the polar units, but the opposite was observed. At this point, the most plausible hypothesis is that the amine-derived segments disturbed the packing of the linear PEO chains, increasing the free space, and thus facilitating mobility. However, this needs to be clarified in future work.

Another challenging observation regarding the dynamics of the two systems is that, based on the Angell plots (Figure 11), the PEO-based system had an identical degree of cooperativity (fragility) as that of the system based on a PPO chain with a ~40% lower molar mass, and therefore a higher concentration of amine-derived units. Meanwhile, the system based on a higher *M_w_* PPO showed much less cooperativity. This observation is consistent with the assumption of more free volume in the PEO-based system [83].

The ratio of bulky ‘characteristic’ amine units over short aliphatic amine units did not have a monotonic influence on the dynamics. Similar observations have been made in a previous work using the ratio of units bearing secondary amine groups to simple aliphatic units [57]. However, in both cases, the influence on dynamics was an indirect effect controlled by hydrogen bonding between carbonyl units on the amide group and other units of the chain.

The chemical structure of the characteristic amine units also influenced segmental mobility. This can happen through a multitude of mechanisms. A direct mechanism involves the mobility of the amine itself. Bulky, rigid molecules have lower mobility, which should be reflected in their copolymers in regard to the mixing laws mentioned above [54,55,56,84,85]. However, we observed here that the bulkiest of the amines (P74), which also caused the strongest hydrogen bonding, created the system with the lowest *T_g_* among the studied amines. This brings forward a second direct mechanism, namely the effect of free volume, which in this case was introduced by the long side chains that were added to the system [86]. Moreover, in the P74 amine, the cycloaliphatic ring is far from the urethane units, giving the system more degrees of freedom. Besides these direct methods, hydrogen bonding, as mentioned earlier, has a strong influence on the glass transition temperature (Ref. [57] and references therein). This is possibly the reason why the linear, rather flexible, but hydrogen donor-containing TETA slowed down the mobility, similar to the other bulkier amines. It should be noted, however, that the fact that TETA is linear helps with tighter packing (less free volume), which was corroborated by the higher fragility it exhibited compared to its bulkier counterparts (Figure 11).

The dynamic glass transition generally corresponds very well with the thermal one. In the Arrhenius graphs in Figure 10, it is clearly visible that the relaxation traces of α relaxation corresponded well with the calorimetric glass transition temperatures plotted at an equivalent relaxation time of 100 s. However, the traces have an unusual behavior: there was a very subtle change in curvature around the kHz region for PPO-based materials corresponding to 70–80 °C and for PEO-based materials around 50 °C. The origin of this effect is not clear at this point. It was not accompanied by visible events either on the DSC curves (Figure 3) or in the isothermal plots of *ε*′ (Figure 5). Hence, it should be attributed either to a structural transition, with a very weak effect on heat capacity and polarizability, or to a dynamic phenomenon. One hypothesis is that it might be related to the phenomenon of dynamic transcarbamoylation, which is known to be present in polyhydroxyurethanes [87,88]. Transcarbamoylation is a reversible reaction between hydroxyl and urethane groups, which, in PHUs, can lead to the formation of a dynamic network. However, more research is needed on this matter.

An interesting observation is that the charge mobility was fully controlled by segmental dynamics. The connection between the time scale of α relaxation (dynamic glass transition), as expressed by the characteristic frequency of the Havriliak–Negami model, and the charge mobility expressed by the dc conductivity followed a power law. However, strikingly, all traces, irrespective of the type of amine, composition, or polyether, collapsed into the same master curve. More interestingly, the exponent of the power law had good accuracy, which was equal to unity. The reason behind this observation is yet another point worth studying in future work.

## 4. Materials and Methods

### 4.1. Materials

All reagents, catalysts, and solvents mentioned in Section 4.2. were purchased from Sigma (Piekary Śląskie, Poland) and were used as received.

### 4.2. Synthesis

The synthesis of polyhydroxyurethanes was carried out in two steps in a manner very similar to that reported in Ref. [57]: (i) formation of prepolymer and (ii) chain extension.

Each system was synthesized with (a) an oligoether-based bifunctional cyclic carbonate (PPO with a molar mass of 400 or 650, or PEO with a molar mass of 700); (b) a diamine named in the context of this article “characteristic” (TETA, MBCA, IPDA, or P74; Table 1); and (c) a short linear diamine, that is, diaminobutane (DAB). On the basis of the nominal molar masses of the reagents, their amounts for each synthesis reaction were calculated beforehand so that (a) the final polymer mass was 5 g; (b) the amine component (characteristic amine + diaminobutane (DAB)) was equimolar to the CC; and (c) the molar ratio of the CC to the characteristic amine was the one provided in Table 1.

**Figure 14:**
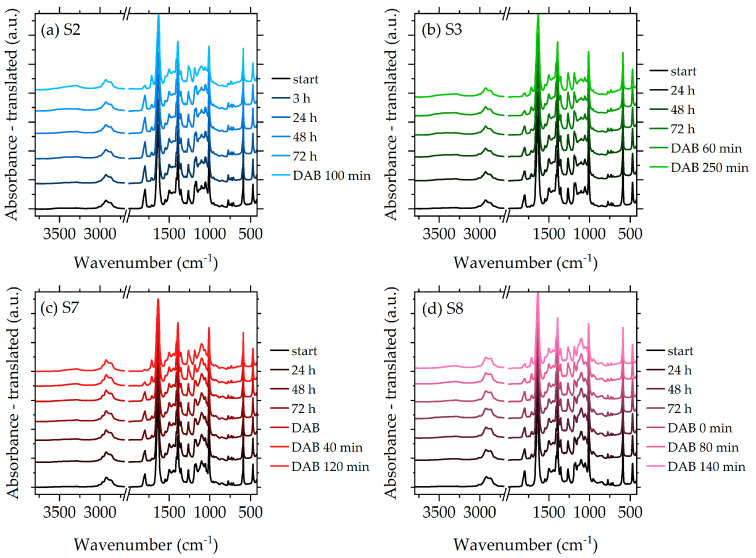
FTIR spectra of the reaction mixtures at different reaction times for samples (**a**) S2, (**b**) S3, (**c**) S7, and (**d**) S8 as representative specimens.

In the first step, the cyclic carbonate (PPO with a molar mass of 400 or 650, or PEO with a molar mass of 700), the characteristic amine, the catalyst (TBD, 5 mol% with respect to the sum of the moles of the reagents), and the solvent (DMAc, 15 mL) were added to a three-neck flask equipped with a sampling system, magnetic stirrer, and reflux condenser. The reaction was carried out at 75 °C with continuous mixing (300 rpm). The progress of the synthesis was monitored by following the changes in the FTIR spectra (Figure 14) in the region of C=O stretching vibrations of the CC ring (band at 1800 cm^−1^, gradual decrease) and urethane (1700 cm^−1^, gradual increase). When no visible changes were observed in the FTIR spectra between two subsequent measurements with an interval of 30 min, the formation of the prepolymer was considered complete. In the second step, DAB was added as a chain extender in the amount that would yield an equimolar ratio of amine groups to unreacted CC rings. The second step of the reaction was allowed to run until the band associated with the CC rings (1800 cm^−1^) disappeared. The mixture was transferred into a polypropylene mold and conditioned in an oven at 100 °C, under reduced pressure, to remove the solvent.

Before the DSC and BDS experiments, the samples were conditioned overnight in a vacuum oven at 60 °C, and then kept for several weeks in a desiccator over phosphorous pentoxide to remove any residual humidity.

### 4.3. Fourier Transform Infrared Spectroscopy (FTIR)

Infrared spectroscopy experiments were performed using a Thermo Scientific Nicolet iS5 spectrometer equipped with the iD7 Attenuated Total Reflectance (ATR) Accessory. The wavenumber range was 4000–400 cm^−1^ with a scanning resolution of 4 cm^−1^. A total of 16 scans were averaged for each measurement.

### 4.4. Differential Scanning Calorimetry (DSC)

Differential scanning calorimetry experiments were carried out using a Mettler Toledo 822e calorimeter purged with argon and cooled with liquid nitrogen. Samples with weights of 6 to 7 mg were previously dried for several weeks over P_2_O_5_. The measurement consisted of a single scan from −70 to 200 °C at a rate of 10 K/min. The reported glass transition temperatures (*T_g_*) are the midpoint values.

### 4.5. Broadband Dielectric Spectroscopy (BDS)

The dielectric function $\varepsilon^{*}\left(f\right)=\varepsilon^{\prime }\left(f\right)-i\varepsilon^{\prime \prime }(f)$ spectra were recorded isothermally in temperature steps of 5 or 10 K from −100 °C up to 90 °C, in the frequency range of 100 mHz–1 MHz, and with a voltage amplitude of 1.0 V. The samples were films with a thickness of 0.5–1.0 mm and diameter of ~10 mm, and were previously dried for several weeks over P_2_O_5._ Brass electrodes with a diameter of 9.6 mm were used for the measurements. The spectra were recorded using a Turnkey Impedance Spectrometer Concept 81 (Novocontrol Technologies GmbH & Co. KG, Montabaur, Germany). The accuracy of the temperature control was greater than 0.5 K.

Due to the high dc conductivity of the samples, for the study of α relaxation, a ‘conductivity-free’ imaginary part of the dielectric function *ε*″_*der*_ was approximated using an approach proposed by Wübbenhorst and Van Turnhout [78].

(2) $$\varepsilon_{der}^{\prime \prime }(f)=-\frac{\pi }{2}\frac{\partial \varepsilon^{\prime }(\omega )}{\partial \ln\omega }$$

where ω=2πf is the angular frequency of the field.

The spectra were analyzed by fitting model functions on them; the most general one is the Havriliak–Negami one; for a single relaxation, it is expressed as [80,89,90]

(3) $$\varepsilon^{*}\left(f\right)=\varepsilon^{\prime }\left(f\right)-i\varepsilon^{\prime \prime }\left(f\right)=\varepsilon_{\infty }+\frac{∆\varepsilon }{{\left(1+{\left(i\frac{f}{f_{0}}\right)}^{a}\right)}^{b}}$$

where ∆ε is the contribution of a relaxation to the total dielectric permittivity of the material, and $f_{0}$ its characteristic frequency. The exponent a≤1 expresses a symmetric broadening in the frequency domain with respect to the Debye single relaxation time model, which has an a=1. Exponent b expresses an asymmetric broadening on the high-frequency side of the relaxation. b=1 expresses a symmetric relaxation, and the equation reduces to the Cole–Cole equation [91]. $\varepsilon_{\infty }$ corresponds to instantaneous polarization, i.e., the contributions of all faster relaxations to the dielectric permittivity of the material.

The peak frequency $f_{max}$ of a Havriliak–Negami relaxation, as a function of the model parameters, was calculated using [92]

(4) $$f_{\max}=f_{0}{\left(\frac{\sin\left(\frac{a}{1\;+\;b}\;\frac{\pi }{2}\right)}{\sin\left(\frac{ab}{1\;+\;b}\;\frac{\pi }{2}\right)}\right)}^{\frac{1}{a}}$$

In the case b=1, $f_{max}$ coincides with $f_{0}$.

A sum of two such Cole–Cole terms, one for each secondary relaxation, was fitted on the $\varepsilon^{\prime \prime }\left(f\right)$ spectra in the region of secondary relaxations. A model based on Equation (1) with $\varepsilon^{\prime }(f)$ calculated from Equation (2) was fitted on the $\varepsilon_{der}^{\prime \prime }(f)$ spectra in the region of α relaxation.

The activation energies for secondary relaxations were calculated by fitting the Arrhenius equation

(5) $$f_{max}=f_{p}e^{-\frac{E_{act}}{kT}}$$

onto the $\log\left(f_{max}\right)$ vs. $\frac{1}{T}$ data (Arrhenius plot).

The complex conductivity of the samples was calculated as [80]

(6) $$\sigma^{*}\left(f\right)=i2\pi f\varepsilon_{0}\varepsilon^{*}(f)$$

where *ε*_0_ is the permittivity of the free space.

The dc conductivity $\sigma_{dc}$ was calculated as the plateau value of the $Re(\sigma^{*}\left(f\right))$ spectra at frequencies lower than the α relaxation but higher than the onset of electrode polarization.

## 5. Conclusions

The molecular mobility of polyhydroxyurethanes can be tailored through the proper selection of both cyclic carbonate and amine components. The dynamic and calorimetric studies consistently showed the same dependencies.

The factor that influences the molecular mobility the most seemed to be the spacing between hydroxyurethane moieties. Upon increasing the molecular weight of the cyclic carbonate, the glass transition temperature decreased significantly. As the cyclic carbonate was the main mass contributor in the materials studied, it was also expected that changes in the structure of the CC chain will influence the mobility, and indeed, this was the case.

The cyclic carbonate based on the more mobile PEG oligomer exhibited notably higher mobility in comparison to the systems based on the PPG oligomeric cyclic carbonate.

Although amine components constituted only a small part of the mass in the final material, their structure also influenced the mobility, though to a lesser extent. Cyclo-aliphatic structures and groups acting as donors/acceptor of hydrogen bonding in the amine structure slowed down the molecular mobility of the system. An increase in the rigidity of the polymer chain affected mobility to a larger extent than the formation of additional hydrogen bonds. This should be attributed to the already dense hydrogen bonding in polyhydroxyurethanes. 

The charge mobility was strongly coupled with the segmental mobility, with all materials following the same power law, irrespective of their structure. The reason behind this observation is worth studying in more depth in future work.

Secondary relaxations are less affected by the macrochain structure, mostly due to their local character. Nevertheless, it can be stated that the relaxation related to the urethane unit (β relaxation) was more sensitive to structure than the one related to the non-polar backbone of the polyether. This could be related to the fact that the urethane group is able to interact, to some extent, with the moieties around it. In summary, our work showed that the glass transition, both calorimetric and dynamic, in polyhydroxyurethanes is controlled by a multitude of parameters and mechanisms. This is true also for conventional polyurethanes, where, although each system needs to be addressed depending on its own particularities, the principles are quite well known. However, understanding the glass transition in non-isocyanate polyhydroxyurethanes, especially in conjunction with charge mobility and other phenomena that may be present, remains a thrilling path to explore.

## Figures and Tables

**Table 1:** Overview of all materials under investigation: components, molar ratios, glass transition temperatures *T_g_*, and heat capacity changes Δ*c_P_*_._

Code	Characteristic Amine (CA)	Polyether CC (P-CC)	Molar Fraction (P-CC:CA)	Group(s)	*T_g_* (°C) (±1.0 °C)	Δ*c_P_* (J/gK) (±0.05 J/gK)
S1	Priamine 1074 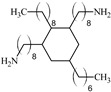	PPO *M_w_* ~460 g/mol 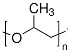	3:1	I	10.8	0.63
S2	IPDA 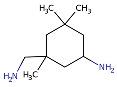				18.0	0.56
S3	TETA 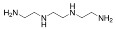				14.6	0.59
S4	MBCA 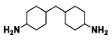			I and II	15.9	0.57
S5			2.5:1	II	16.9	0.51
S6			2:1	II and III	14.1	0.57
S7		PPO *M_w_* ~700 g/mol 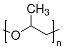		III	−7.6	0.57
S8		PEO *M_w_* ~650 g/mol 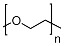			−21.7	0.67

**Table 2:** Strength parameter Δ*ε* and Havriliak–Negami exponents for all materials under investigation at the characteristic temperature of 65 °C. Reported inaccuracies are standard errors estimated by the lmfit routine of scipy.

Code.	(CA)	(P-CC)	Molar Fraction P-CC:CA	Group	α Relaxation		
					Δ*ε*	*a*	*b*
S1	P74	PPO 460	3:1	I	30.9 ± 0.2	0.68 ± 0.01	0.55 ± 0.02
S2	IPDA				40.9 ± 0.4	0.63 ± 0.01	0.71 ± 0.02
S3	TETA				26.1 ± 0.2	0.60 ± 0.01	0.64 ± 0.02
S4	MBCA			I, II	27.5 ± 0.2	0.67 ± 0.01	0.54 ± 0.02
S5			2.5:1	II	20.7 ± 0.2	0.65 ± 0.01	0.53 ± 0.02
S6			2:1	II, III	32.4 ± 0.7	0.64 ± 0.02	0.67 ± 0.03
S7		PPO 700		III	20.5 ± 0.3	0.66 ± 0.03	0.53 ± 0.05
S8		PEO 650			20.2 ± 0.8	0.79 ± 0.03	0.30 ± 0.04

## Data Availability

Datasets are available on request from the authors.
